# Apathy and Anhedonia: Clinical and Neurophysiological Assessment of a Romanian Cohort

**DOI:** 10.3390/brainsci11060729

**Published:** 2021-05-31

**Authors:** Diana Sipos-Lascu, Ștefan-Cristian Vesa, Lăcrămioara Perju-Dumbravă

**Affiliations:** 1 Department of Neurosciences, “Iuliu Hațieganu” University of Medicine and Pharmacy, 400012 Cluj-Napoca, Romania; lperjud@umfcluj.ro; 2 Neurology I Clinic, Cluj-Napoca Emergency Clinical County Hospital, 400012 Cluj-Napoca, Romania; 3 Department of Pharmacology, Toxicology and Clinical Pharmacology, “Iuliu Hațieganu” University of Medicine and Pharmacy, 400337 Cluj-Napoca, Romania; stefanvesa@gmail.com

**Keywords:** Parkinson’s disease, apathy, anhedonia, visual evoked potentials

## Abstract

Background: Patients with Parkinson’s disease (PD) often have, besides the characteristic motor manifestations, a wide variety of non-motor symptoms. These include apathy and anhedonia, common issues in PD, which can be quantified with the help of evaluation scales recommended by the literature. There are sensory non-motor manifestations of PD, some of which are easy to detect through electrophysiological studies. Our aim was to investigate the possible association of apathy and anhedonia with the severity of the motor status in a sample of PD patients in Romania. We also examined the prevalence of latency changes in the P100 wave of visual evoked potentials (VEPs) and how they correlated with motor status, apathy, and anhedonia in PD patients. Methods: Thirty-four patients with PD participated in this study. All were assessed for motor status using the Unified Parkinson’s Disease Rating Scale (UPDRS) and were rated on the Hoehn and Yahr scales. The presence and severity of apathy and anhedonia were assessed using the Apathy Evaluation Scale (AES), the Dimensional Apathy Scale (DAS), the Lille Apathy Rating Scale (LARS), and the Snaith–Hamilton Pleasure Scale (SHAPS). The latency of the P100 wave of the VEP was measured in all the patients. Results: Apathy and anhedonia were common among the patients with PD (35% and 58.8%, respectively). The presence of apathy/anhedonia was correlated with the severity of motor symptoms, as assessed using the UPDRS scale (*p* < 0.001), and with the stage of the disease according to the Hoehn and Yahr scale (*p* < 0.001). A prolonged latency of the P100 wave of the VEP was observed among apathetic (*p* < 0.001)/anhedonic (*p* < 0.01) patients and those with increased disease severity (*p* < 0.001). Conclusion: Apathy and anhedonia are common in PD and may correlate with the severity of motor symptoms. There may be visual impairment in these patients, evidenced by a prolonged P100 latency, which correlates with the severity of disease. Significance: Scales for assessing apathy and anhedonia, as well as measuring VEP latency, could be useful in assessing the severity of disease.

## 1. Introduction

Apathy is one of the most common non-motor disorders in Parkinson’s disease (PD), with a reported prevalence varying from 17% to 60% between different studies [1,2]. It has been defined as a reduction in motivation, simultaneously affecting the behavioral, cognitive, and affective spheres [1,3]. In half of the cases, it occurs alone, without depression or cognitive impairment [1]. It can appear from the beginning of the disease, or it may occur during the course of the disease, constituting a negative prognostic factor [4]. Apathy was associated with a lower score on the Mini Mental State Examination scale, a higher disease stage on the Hoehn and Yahr scale, and a higher UPDRS score [1,5,6].

Anhedonia, defined as an inability to feel joy and pleasure, an entity also common among patients with PD, has been associated with a poorer motor status [7,8]. Some studies have considered anhedonia to be a manifestation of depression or apathy [7,8]. Other authors have claimed that there is no link between anhedonia, depression, and apathy, but they observed a much higher frequency of anhedonia in PD patients than in a control group [9]. Several scales for identifying and assessing the severity of apathy and anhedonia have been proposed by the existing literature (AS, AES, LARS, LARS, and DAS for apathy; SHAPS and the Chapman scale for anhedonia), some of which have already been validated in PD [5,6]. Apathy and anhedonia in PD have also been correlated with sensory dysfunctions, particularly visual abnormalities, objectified with the help of electrophysiological studies [10]. In PD patients, the latency of N75, P100, and N145 waves was significantly prolonged, especially in the more advanced stages of the disease. The amplitude of the P100 wave did not change [10,11,12,13,14]. Moreover, some authors observed a difference in the latency of the P100 wave between the two eyes, with greater magnitude among PD patients than that in the control group [13].

The aim of this study was to investigate the impact that affective non-motor symptoms (apathy and anhedonia) have on the motor status of PD patients in Romania. Moreover, we aimed to determine whether there was an association between retinal dopaminergic cell degeneration (evidenced by prolonged VEP latency) and loss of dopamine in the reward system (evidenced by apathy/anhedonia).

The main hypothesis of our study states that PD patients who develop non-motor symptoms such as apathy/anhedonia during the evolution of disease present more severe motor symptoms than those who do not have these non-motor manifestations. In addition, VEPs have longer latencies in patients with more severe motor (higher UPDRS and Hoehn and Yahr stage) and non-motor (apathy/anhedonia) symptoms. 

## 2. Materials and Methods

The current study wasa cross-sectional, observational study and enrolled 34 patients with Parkinson’s disease who presented to the Neurology I Clinic, Cluj-Napoca County Emergency Clinical Hospital, during the period of 1 October 2019–15 January 2021. 

The inclusion criteria were as follows: patients with Parkinson’s disease, Hoehn and Yahr stage 1–3 with or without antidepressant therapy, and the provision of signed documentation providing informed consent for voluntary participation in the study. Patients with mourning reactions and those who did not sign the informed consent form for participating in the study and/or the agreement regarding personal data processing for research purposes were excluded. The study was approved by the ethics committee of the “Iuliu Hațieganu” University of Medicine and Pharmacy, Cluj-Napoca, Romania (registration number: 90/2021). 

The anamnestic data of each patient included were collected, and neurological examinations were performed, each of which was followed by the calculation of the UPDRS score and placement on the Hoehn and Yahr scale. All the patients underwent psychological evaluation, including on the MMSE scale. Psychiatric examinations were performed on patients for whom the psychological evaluations highlighted depressive elements, increased emotional reactivity, moderate/severe anxiety (according to the Leahy anxiety scale), or moderate/severe cognitive impairment (according to MMSE). The patients were rated using apathy scales (the Lille Apathy Rating Scale, Apathy Evaluation Scale, Dimensional Apathy Scale, and UPDRS part I item 4) and anhedonia scales (Snaith–Hamilton Pleasure Scale). Cut-off values determined in validation studies for these scales were used.

Patients who met the inclusion criteria underwent VEP, using the same potentially evoked response unit (Keypoint 4, Medtronic, Denmark; software: Keypoint v. 5.11- Alpine BioMed) through the “Reversal Pattern” technique. The reversal rate was 2 Hz.

The test was performed on each eye separately on each subject, the other eye being covered during the test. The latencies of waves N75, P100, and N135 and the amplitude of the P100 wave were recorded.

All of our scores and neurophysiological tests were performed while patients with motor fluctuations were in the “on” phase. 

Statistical analyses were performed using the MedCalc Statistical Software version 19.6.4 (MedCalc Software Ltd., Ostend, Belgium; https://www.medcalc.org). Quantitative data are expressed as medians and 25th–75th percentiles (non-normally distributed according to the Shapiro–Wilk test). Qualitative data are expressed as frequencies and percentages. Comparisons between groups were performed using the Mann–Whitney U test. The correlations between quantitative variables were verified using Spearman’s rho. A *p* value < 0.05 was considered statistically significant.

Our aim was to investigate the association of apathy and anhedonia with the prevalence of latency changes in the P100 wave of the VEP. We also examined how this correlated with the severity of the motor status.

## 3. Results

### 3.1. Demographic Data

We collected patient demographic data. These are presented together with specific scores applied in Table 1.

### 3.2. Statistical Analysis

The disease scores and apathy/anhedonia scores were correlated (Table 2). The UPDRS scores were strongly correlated with all the apathy scores, anhedonia scores, and doses of levodopa. The Hoehn and Yahr stage was strongly correlated with the LARS/DAS scores and moderately correlated with the AES score.

We found a strong correlation between a prolonged VEP latency and the presence of apathy, anhedonia, and a higher levodopa dose (Table 3). 

We observed several associations between the disease scores and the presence of certain symptoms (Table 4). Patients with apathy or depression had statistically significantly higher Hoehn and Yahr stages and higher UPDRS scores. This was also observed in patients with prolonged PEV. Patients with anhedonia had significantly higher UPDRS scores.

## 4. Discussion

In addition to the pathophysiological changes that define PD (the degeneration of dopaminergic neurons in the substantia nigra pars compacta), there is a progressive concomitantdegeneration of noradrenergic neurons in the locus coeruleus and acetylcholine cells, changes that may be responsible for the onset and worsening of affective non-motor symptoms, such as apathy and anhedonia [15]. Thus, regulating dopaminergic transmission through the serotonergic system in the areas of the brain involved in the reward process may explain why previous studies found dopaminergic therapy to have a reduced effect on anhedonia [15]. Some authors support the hypothesis that the anhedonia in PD may be due to a predominant inhibition of D2 dopaminergic receptors, which may explain its improvement in some patients treated with certain dopaminergic agonists, such as pramipexole [16,17]. In our study, we found a frequency of apathy of over 35% in the group enrolled, consistent with the existing literature, and an anhedonia frequency of almost 59% (identified using the SHAPS scale), a value higher than the values stated in the previous literature [1,2]. Most patients with apathy (66.7%) had a previously established diagnosis of depression, while half of the anhedonic patients did not present depression. Previous studies concluded that, even though apathy can be associated with anhedonia and fatigue, half of the PD patients with apathy do not experience depression, apathy being considered a separate entity in these patients [3]. Several studies found anhedonia to be closely related to depression [17,18], while other authors indicated that it was an independent phenomenon [9,18]. Anhedonia is also one of the key symptoms of major depressive disorder [19,20]. Our patients with higher UPDRS scores had higher apathy and anhedonia scores and took higher doses of levodopa. Subjects in more advanced stages of the disease, according to the Hoehn and Yahr classification, had higher scores on the LARS and DAS apathy scales than those in the early stages. In previously published studies, apathy among PD patients was associated with older age, a higher score on the UPDRS scale, a lower MMSE score, an increased risk of developing concomitant depression, and a higher degree of disability [1]. Apathy has been considered an independent entity in PD, attributable to the degenerative disease itself, given that half of cases in a study did not suffer from concomitant depression or cognitive dysfunction [1]. Some authors have concluded that, in the early stages of PD, among patients who have never received dopaminergic treatment, apathy is associated with poorer motor status, impaired cognitive function, and a poorer quality of life [3]. The degeneration of neurons in reward centers or areas responsible for goal-oriented behavior has been implicated in the onset of apathy in Parkinson’s disease [21]. For the identification and assessment of the severity of apathy, several scales have been used in previous studies (the Apathy Scale, the Apathy Evaluation Scale, the Lille Apathy Rating Scale, the Dimensional Apathy Scale, and the Apathy Inventory) [20,22]. Part 1 item 4 of the Unified Parkinson’s Disease Rating Scale and item 7 of the Neuropsychiatric Inventory have been considered useful by some authors for screening [20,22]. Some of these scales were used in the current study.

Regarding anhedonia, common among the population with PD, some authors have observed a correlation between the presence/severity of anhedonia and the severity of motor manifestations, the degree of restriction in daily activity, and depression in these patients [23]. Anhedonia has also been considered one of the main features of melancholy, a type of depression common in patients with PD [17,24]. On the contrary, other authors have disputed the existence of any correlation between anhedonia, disease duration, and motor status in patients with PD [9]. A decrease in the level of dopamine, the essential neurotransmitter in the reward system, has also been implicated [16,17,25]. To identify anhedonia and assess its severity, previous studies have used the SHAPS and Chapman scales [20]. The first was also suggested for evaluating the response to pharmacological treatment, being sensitive to changes in hedonic tone [20,23,26,27,28,29,30].

The prevalence of anhedonia (assessed on the SHAPS scale) among our patients (58.8%) was considerably higher than that in previous published studies (up to 46%), probably due to a large proportion of patients with increased disease severity [17]. However, we believethat the epidemiological situation in which most of the study took place (the SARS-CoV-2 pandemic) and the restrictive measures imposed in our country to prevent the spread of the virus during the emergency/alert state were important in this regard. Given that there are no other previous studies quantifying anhedonia in PD patients during a pandemic, we could not compare this result with others obtained in a similar context. We used the SHAPS scale in its original version, in English, because it has not yet been validated in the native language of the authors (Romanian).

Almost all the patients with apathy (91.7%) also had anhedonia, while more than half of the anhedonic patients (55%) had apathy. These data are in accordance with those presented in the existing literature, which can probably be explained by the common mechanism of occurrence of the two non-motor manifestations [17].

The presence of alpha-synuclein neuronal aggregates is a feature of PD [31]. A distribution of these aggregates was found at the level of the inner nuclear layer (INL) and the inner plexiform layer (IPL) of the retinal structure, a fact that could explain the disturbances objectified by the latency changes of the VEP in PD [32]. The distribution of alpha-synuclein in the retinas of PD patients was different from that seen in patients with Lewy body dementia or Alzheimer’s disease or in the elderly population [32]. These findings are consistent with the clinical and neurophysiological manifestations of visual disturbance in PD [32]. 

A number of studies have shown a significant slowing of the N75, P100, and N145 waves of the reversal pattern VEP in patients with Parkinson’s disease [10,33]. The amplitude of the P100 wave is not changed [10]. The VEP can illustrate the integrity of the entire visual pathway, its abnormalities in PD patients being able to show that the widespread characteristic biochemical disorders also affect the retina [10]. A possible mechanism could be the degeneration of amacrine dopaminergic cells in the retina [11,12]. The latencies of the VEP components were greater in patients with PD and correlated with motor status and medication rather than with cognitive function [34]. Because basal ganglia—whose function is regulated by dopamine—plays an essential role in the occurrence of motor symptoms in PD patients, some authors considered that evoked potentials reflect the function of basal ganglia [35]. The N75 and P100 components of the VEP are considered to have their origins in the visual cortex, while the N145 component originates from the extrastriate cortex [35,36]. Based on central conduction times, differences between PD patients and healthy controls were not observed, and some studies support the idea that the origin of the conduction delay is peripheral rather than cortical [4,11]. Regarding motor symptoms, both the total UPDRS score and the score of UPDRS part 3 assessed in the on state were positively associated with the latency of the P100 component [35]. The P wave latency increased with the progression of the disease [13]. In addition, there was a greater latency difference between the two eyes among patients with PD compared to the control group [13].

In our study, we observed a statistically significant correlation between the P100 wave latency and the disease stage according to the Hoehn and Yahr scale and the UPDRS score. Patients with prolonged P100 wave latencies had higher UPDRS scores than those with unmodified VEP and more severe stages of the disease according to the Hoehn and Yahr scale. This can be explained by the progressive degeneration of dopaminergic cells in the retina, which appears simultaneously withpathophysiological changes that define Parkinson’s disease (the degeneration of dopaminergic neurons in the substantia nigra pars compacta) [37]. Most apathetic patients (83.3%) and almost half of the anhedonic patients (45%) had prolonged latencies of the P100 wave of visually evoked potentials. To our knowledge, there are currently no other published studies comparing prolonged VEP latencies (probably explained by retinal dopaminergic cell degeneration) and psycho-affective manifestations, such as apathy/anhedonia (a loss of dopamine in the reward system). However, there have been many electrophysiological studies in PD patients. Some used P100 wave latency measurements to assess visual pathwayimpairment in PD patients, regardless of the presence of affective symptoms, while others investigated the relationship between apathy and attention deficit disorder, using the P300 wave of human-event-related potentials [37].

Some studies observed a normalization of wave latency after levodopa treatment, while others showed no change [12,14,34,38].

The latency of the P100 wave is considered a sensitive measure of changes in PD, as it is little influenced by dopaminergic treatment [10]. Studies on evoked responses in PD patients have proved to be useful for elucidating the etiology and quantitative evaluation of PD [35].

Our study has several limitations: (1) the cohort used comprises only 34 patients with Parkinson’s disease, without a control group; (2) the vast majority of the patients were included and evaluated during the SARS-CoV-2 pandemic, so the global epidemiological situation, as well as the state of emergency/alert established in Romania, could have exacerbated the patients’ psycho-affective symptoms; (3) the scales used to assess psycho-affective manifestations were applied in their original version, in English, because they have not yet been validated in Romanian; and (4) we could not quantify the impact that dopaminergic therapy had on the psycho-affective symptoms of the enrolled patients, because almost all of them were already under treatment.

## 5. Conclusions

Apathy and anhedonia often occur in patients with PD. They may overlap with depression or can appear as individual phenomena. Several scales can be used to detect and measure apathy (AES, DAS, and LARS) and anhedonia (SHAPS) quickly and easily in PD patients with or without depression.

By measuring the VEP latencies, we can assess the integrity of the visual pathway, often modified in the evolution of the disease. The changes observed in the measurement of VEPs were correlated with the scores obtained on the apathy and anhedonia scales. A prolonged latency of the P100 wave of the VEP has also been associated with disease severity.

We believe that these data could be useful in the future for a more accurate quantification of the real severity of Parkinson’s disease and for the earliest therapeutic approach to all its implications.

## Figures and Tables

**Table 1:** Demographic and neurologic data.

Variable		Characteristic
Age (years)		65.8 ± 7.8 *
Gender	Male	19 (55.9%) **
	Female	15 (44.1%) **
Medium of origin	Rural	3 (8.8%) **
	Urban	31 (91.2%) **
Hoehn and Yahr		4 (3; 5) ***
UPDRS		34.5 (20.5; 43.25) ***
UPDRS part I item 4		0 (0; 1) ***
MMSE		29 (27; 29) ***
LARS		−18.5 (−26.25; −4.50) ***
DAS		7 (5.75; 15) ***
AES		22.5 (15; 38.25) ***
SHAPS		21 (16; 48) ***
Apathy		12 (35%) **
Anhedonia		20 (58.8%) **
Anhedonia in apathic patients		11 (91.7%) **
Apathy in anhedonic patients		11 (55 %) **
Depression	Apathy	8 (66.7%) **
	Anhedonia	10 (50%) **
Prolonged VEP	Apathy	10 (83.3%) **
	Anhedonia	9 (45%) **

* mean and standard deviation; ** frequency and percentage; *** median and 25th and 75th percentiles.

**Table 2:** Correlations between disease scores and apathy/anhedonia scores.

Variable	Hoehn and Yahr		UPDRS		UPDRS Part I Item 4	
	r	*p*	r	*p*	r	*p*
LARS	0.621	<0.001	0.741	<0.001	0.825	<0.001
DAS	0.540	<0.001	0.785	<0.001	0.637	<0.001
AES	0.408	0.01	0.713	<0.001	0.813	<0.001
SHAPS	0.309	0.07	0.560	<0.001	0.541	<0.001
Levodopa dose	0.797	<0.001	0.551	<0.001	0.642	<0.001

**Table 3:** Correlations between prolonged visual evoked potentials and presence of apathy/anhedonia/levodopa dose.

	Apathy	Anhedonia	Levodopa Dose
Prolonged VEP	*r* = 0.84 *p* < 0.001	*r* = 0.409 *p* = 0.016	*r* = 0.609 *p* < 0.001

**Table 4:** Associations between disease scores and several variables.

Variable		Hoehn and Yahr Scale		UPDRS		UPDRS Part I Item 4	
		Median (25th–75th Percentiles)	*p*	Median (25th–75th Percentiles)	*p*	Median (25th–75th Percentiles)	*p*
Apathy	Yes	5 (5; 5)	0.001	47 (41.5; 55.75)	0.000	1 (1; 2.75)	0.000
	No	3.5 (3; 5)		29 (16; 36)		0 (0; 0)	
Anhedonia	Yes	5 (3.25; 5)	0.197	40 (29.25; 48.75)	0.011	1 (0; 1)	0.043
	No	4 (3; 5)		29.5 (14.75; 29.25)		0 (0; 1)	
Depression diagnosis	Yes	5 (4; 5)	0.046	40.5 (31.75; 47.5)	0.011	1 (0; 1)	0.068
	No	3.5 (3; 5)		27.5 (16; 39.25)		0 (0; 1)	
Dopaminergic agonist	Yes	3 (3; 5)	0.061	29 (16; 35.5)	0.041	0 (0; 0.5)	0.038
	No	5 (4; 5)		39 (32; 45)		1 (0; 1)	
VEP	Normal	4 (3; 5)	0.002	29.5 (16.75; 39)	<0.001	0 (0; 0.75)	<0.001
	Prolonged	5 (5; 5)		47 (42.5; 51.25)		1 (1; 3)	

## Data Availability

Not applicable.
